# Visual Outcomes, Quality of Vision, and Quality of Life of Diffractive Multifocal Intraocular Lens Implantation after Myopic Laser In Situ Keratomileusis: A Prospective, Observational Case Series

**DOI:** 10.1155/2017/6459504

**Published:** 2017-01-04

**Authors:** John S. M. Chang, Jack C. M. Ng, Vincent K. C. Chan, Antony K. P. Law

**Affiliations:** Department of Ophthalmology, Hong Kong Sanatorium and Hospital, Hong Kong

## Abstract

*Purpose*. To report visual performance and quality of life after implantation of a bifocal diffractive multifocal intraocular lens (MIOL) in postmyopic laser in situ keratomileusis (LASIK) patients.* Methods*. Prospective, observational case series. Patients with prior myopic LASIK who had implantation of Tecnis ZMA00/ZMB00 MIOL (Abbott Medical Optics) at Hong Kong Sanatorium and Hospital were included. Postoperative examinations included monocular and binocular distance, intermediate and near visual acuity (VA), and contrast sensitivity; visual symptoms (0–5); satisfaction (1–5); spectacle independence rate; and quality of life.* Results*. Twenty-three patients (27 eyes) were included. No intraoperative complications developed. Mean monocular uncorrected VA at distance, intermediate, and near were 0.13 ± 0.15 (standard deviation), 0.22 ± 0.15, and 0.16 ± 0.15, respectively. Corresponding mean values for binocular uncorrected VA were 0.00 ± 0.10, 0.08 ± 0.13, and 0.13 ± 0.10, respectively. No eyes lost >1 line of corrected distance VA. Contrast sensitivity at different spatial frequencies between operated and unoperated eyes did not differ significantly (all *P* > 0.05). Mean score for halos, night glare, starbursts, and satisfaction were 1.46 ± 1.62, 1.85 ± 1.69, 0.78 ± 1.31, and 3.50 ± 1.02, respectively. Eighteen patients (78%) reported complete spectacle independence. Mean composite score of the quality-of-life questionnaire was 90.31 ± 8.50 out of 100.* Conclusions*. Implantation of the MIOL after myopic LASIK was safe and achieved good visual performance.

## 1. Introduction

With advancements in femtosecond laser and excimer laser, laser in situ keratomileusis (LASIK) has become a common refractive procedure. Over time, all patients who undergo LASIK eventually become presbyopic or cataractous. Given that these patients generally have minimal residual refractive error after LASIK, they are more demanding than the general population regarding the uncorrected vision after intraocular lens (IOL) implantation [1].

Implantation of diffractive multifocal IOLs (MIOLs) provides excellent distance and near vision in patients who undergo refractive lens exchange and cataract surgery [2–11], but the main drawbacks are visual symptoms such as halos and glare [3, 4, 7] and with bifocal MIOLs, poor intermediate vision [3–8]. A recent study [1] reported that patients who underwent cataract surgery after LASIK had more corneal higher-order aberrations (HOAs) than those in the general population. The combined effect of increased corneal and lenticular HOAs may be why they considered cataract surgery earlier than other patients. Since younger patients can accommodate for intermediate visual tasks, this adds an extra risk of dissatisfaction after bifocal MIOL implantation.

Previous studies of eyes after LASIK have focused primarily on monofocal IOLs and the different formulas for IOL power prediction [12, 13]; few studies have reported the visual outcomes of MIOLs after LASIK [14–18]. In those studies, the monocular visual acuity (VA) was often the only main outcome measure to reflect the MIOL performance clinically. The current study reports the monocular and binocular VAs, contrast sensitivity (CS), visual symptoms, spectacle dependence, and quality of life after implantation of a bifocal diffractive MIOL in patients who underwent a previous myopic LASIK procedure.

## 2. Methods

### 2.1. Patients

This prospective, observational case series included patients who underwent unilateral or bilateral implantation of a Tecnis ZMA00 or ZMB00 MIOL (Abbott Medical Optics, Inc., Santa Ana, CA) between July 2009 and November 2014 at the Hong Kong Sanatorium and Hospital after myopic LASIK. The inclusion criteria were age between 40 and 75 years, a preoperative corrected distance VA (CDVA) of 20/40 or better, and a follow-up period of six months or more. The exclusion criteria were an IOL other than the ZMA00 or ZMB00 implanted in either eye, preexisting ocular conditions (e.g., age-related macular degeneration and glaucoma), and systemic diseases (e.g., uncontrolled diabetes mellitus) that may affect the postoperative vision. The ethics committee of our hospital approved the study.

### 2.2. Intraocular Lens

The Tecnis ZMA00 and ZMB00 MIOLs are foldable acrylic diffractive MIOLs with +4 dioptres (D) of near addition. The former has a three-piece haptic design and the latter a single-piece haptic design. The IOLs have a biconvex design with an anterior aspheric surface and a posterior diffractive surface. The overall diameter is 13 mm and the optic diameter is 6 mm. The energy distribution between the distance and near foci is symmetrical and independent of pupillary size [3, 4, 9, 19–21].

### 2.3. Surgical Technique

The same surgeon (J.S.M.C.) performed all surgeries under topical oxybuprocaine 0.4% and intracameral lidocaine 1% or 2%. Preoperatively, the surgeon used nepafenac ophthalmic suspension 0.1% (Nevanac, Alcon Laboratories Inc., Fort Worth, TX) and tropicamide 0.5%-phenylephrine hydrochloride 0.5% (Mydrin-P, Santen Pharmaceutical Co., Ltd., Osaka, Japan) to maintain pupil dilation intraoperatively. A 2.25 or 2.75 mm clear corneal incision was created either superiorly or temporally with a keratome. DisCoVisc ophthalmic viscosurgical device (Alcon Laboratories Inc.) was injected into the anterior chamber and a continuous curvilinear capsulorhexis was created with a pair of forceps. After hydrodissection with or without nuclear splitting, coaxial phacoemulsification was performed using the Infiniti Vision System (Alcon Laboratories Inc.). Irrigation and aspiration of the residual cortex and posterior capsular polishing were performed using a coaxial system. All IOLs were placed in the capsular bag. In patients who underwent femtosecond laser-assisted cataract surgery, anterior capsulotomy and lens fragmentation were performed first with the LensAR laser system (LensAR, Inc., Orlando, FL) before the clear corneal incision was created.

Excimer laser enhancement with the WaveLight Allergetto Wave Eye-Q Laser (Alcon Laboratories Inc.) was performed when the patient was dissatisfied with the residual refractive error. Neodymium-doped: yttrium aluminum garnet (Nd: YAG) laser was performed if there was evidence of posterior capsular opacification that affected vision.

### 2.4. Preoperative and Postoperative Examinations

All patients underwent a preoperative ophthalmologic examination that included noncycloplegic subjective refraction, measurement of CDVA, keratometry, slit-lamp biomicroscopy, and a dilated fundus examination. Corneal topography was measured using the Orbscan IIz (Bausch & Lomb, Rochester, NY) and the Wavelight Oculyzer (Alcon Laboratories Inc.). The axial length and anterior chamber depth were measured using the IOLMaster (Carl Zeiss Meditec AG, Jena, Germany). The clinical history method with the Haigis [22] and SRK/T [23] formulas using the IOLMaster were used for IOL power calculations. The results by the Haigis-L, Modified-Masket, and Shammas formulas from the IOL calculator of the American Society of Cataract and Refractive Society website [24] were also used for supplementary information. The surgeon selected the IOL power at his own discretion. All patients watched a video that showed simulated visual symptoms and were informed about the possibility of permanent visual symptoms.

The postoperative measurements included noncycloplegic subjective refraction, photopic and mesopic pupillary sizes, monocular and binocular uncorrected distance VA (UDVA), CDVA, uncorrected intermediate VA (UIVA) (67 cm), distance-corrected intermediate VA (DCIVA) (67 cm), uncorrected near VA (UNVA) (30 cm), distance-corrected near VA (DCNVA) (30 cm), and CS at spatial frequencies of 3, 6, 12, and 18 cycles per degree (cpd). The intermediate and near vision were measured using the SLOAN Two-Side EDTRS Format Near Vision Chart (Precision Vision, La Salle, IL). The actual VA in logarithm of the minimum angle of resolution at its corresponding distance was calculated by the visual angle subtended for statistical analyses [3, 4, 25]. The CS was recorded using the CSV-1000E (Vector Vision, Greenville, OH). The pupillary size was measured using the Colvard Pupillometer (Oasys Medical Inc., San Dimas, CA). Photopic and mesopic assessments were performed at 85 and 3 candelas/m^2^, respectively.

For consistency, the same research staff administered the National Eye Institute Visual Function Questionnaire-25 (VFQ-25). The questionnaire includes 25 questions grouped into 12 subscales to evaluate quality of life and has been widely used in cataract research [26]. A supplementary questionnaire was also included to document visual symptoms (halos, night glare, and starbursts); satisfaction; spectacle independence (distance, intermediate, and near); whether the patient regretted undergoing surgery; and whether the patient would recommend the surgery to friends or relatives. The patients rated the level of visual symptoms from 0 to 5 (0, none; 1, very mild; 2, mild; 3, moderate; 4, severe; 5, very severe) and satisfaction from 1 to 5 (1, very dissatisfied; 2, dissatisfied; 3, neutral; 4, satisfied; 5, very satisfied).

### 2.5. Statistical Analysis

Statistical analyses included descriptive data for demographics, ocular parameters, visual and refractive outcomes, and questionnaire results. The Kolmogorov-Smirnov test was performed to determine the normality of data. The Friedman test was used to compare the mean absolute error from predicted refraction between IOL formulas (Barrett True K and Barrett True K No History formulas [24] were also used for back calculation), followed by post hoc analysis by Wilcoxon signed-rank tests with Bonferroni correction. Pearson's correlation and linear regression were used to identify a relationship between CS and baseline characteristics, without and with adjustment for other baseline characteristics, respectively. The Mann–Whitney *U* test was used to compare the binocular VA at various distances, level of visual symptoms, satisfaction, and scores of VFQ-25 between patients with unilateral and bilateral implantations. In patients who underwent unilateral implantation, the paired *t*-test and Wilcoxon signed-rank test were used to compare the ocular parameters, refractive outcomes, monocular VA, and monocular CS between the operated and unoperated eyes. *P* < 0.05 was considered statistically significant. All statistical analyses were performed using SPSS version 16.0 (SPSS Inc., Chicago, IL).

## 3. Results

Twenty-three patients (3 men [13%]) were included. Nineteen (83%) and four patients (17%) (mean age, 54.6 ± 4.6 years; range, 49 to 63) underwent unilateral and bilateral implantation, respectively. The mean time from LASIK to MIOL implantation was 10.20 ± 3.44 years (range, 3.99 to 17.30). One eye (4%) underwent femtosecond laser-assisted cataract surgery. Twenty-five ZMA00 (89%) and three ZMB00 (11%) were implanted (mean IOL power, 20.89 ± 3.99 D; range, 5.0 to 28.0). No complications developed intraoperatively. The mean photopic and mesopic pupillary size of the operated eyes were 3.59 ± 0.86 mm (range, 2.50 to 6.00) and 5.81 ± 0.73 mm (range, 4.50 to 7.50), respectively. One eye (4%) required LASIK enhancement for residual mixed astigmatism. Nd:YAG laser was performed in 13 eyes (48%). The mean follow-up period was 904.1 ± 464.8 days (range, 214 to 1,713).

Table 1 shows the baseline characteristics before LASIK and IOL implantation. In patients who underwent unilateral surgery, the baseline characteristics did not differ significantly between the operated and unoperated eyes (*P* > 0.05 for all comparisons).

### 3.1. Refraction

**Figure 1:**
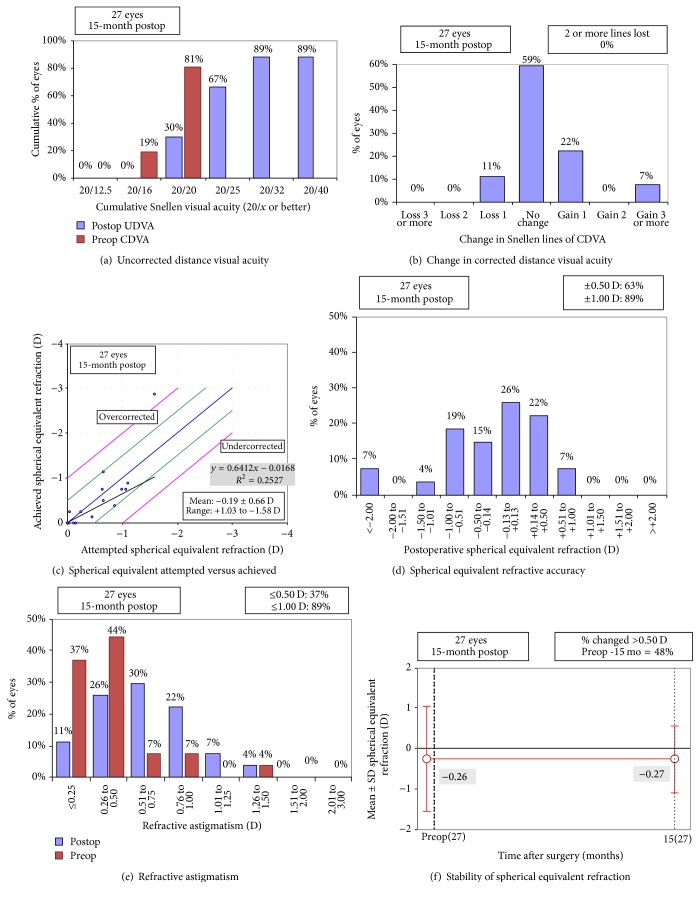
Refractive outcomes, efficacy, and safety (CDVA = corrected distance visual acuity; D = dioptre; SER = spherical equivalent refraction; Postop = postoperative; Preop = preoperative; SD = standard deviation; UDVA = uncorrected distance visual acuity). Haigis-L formula was used in the illustration of spherical equivalent refraction achieved.

The mean postoperative refractive error was −0.64 ± 0.80 D (range, −3.25 to 0.50) sphere and 0.74 ± 0.34 D (range, 0.00 to 1.50) astigmatism with a manifest refraction spherical equivalent (MRSE) of −0.27 ± 0.83 D (range, −2.88 to 0.75).  Figure 1 shows the refractive outcomes.

Twenty-five eyes (93%) with pre-LASIK data available were included for the evaluation of IOL formula accuracy (Table 2). There was a significant difference in the median absolute error between formulas (*P* < 0.001). In the post hoc analysis with Bonferroni correction, the significance level was set at *P* < 0.002. The clinical history method with the Haigis formula showed a mean absolute error of 1.19 ± 0.99 D (range, 0.02 to 5.17) and was significantly larger than that of other formulas (*P* = 0.001 for all comparisons) except for the Modified-Masket (*P* = 0.002) and Shammas formulas (*P* = 0.011). The mean absolute error did not differ significantly between the remaining formulas (*P* ≥ 0.002 for all comparisons), with a range from 0.48 to 0.67 D.

### 3.2. Visual Acuity

**Figure 2:**
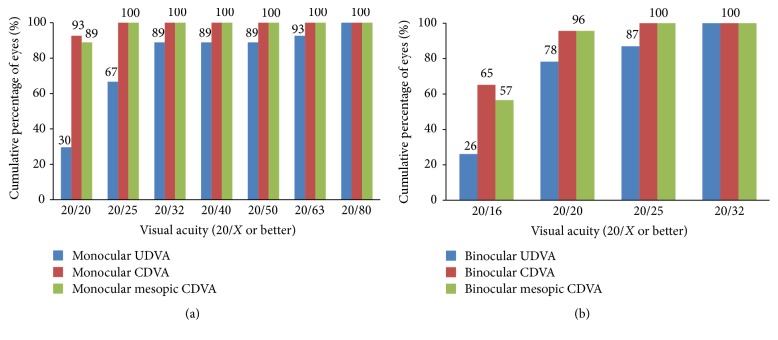
Monocular (operated eyes) and binocular uncorrected distance visual acuity (UDVA), corrected distance visual acuity (CDVA), and mesopic CDVA at the last visit.

**Figure 3:**
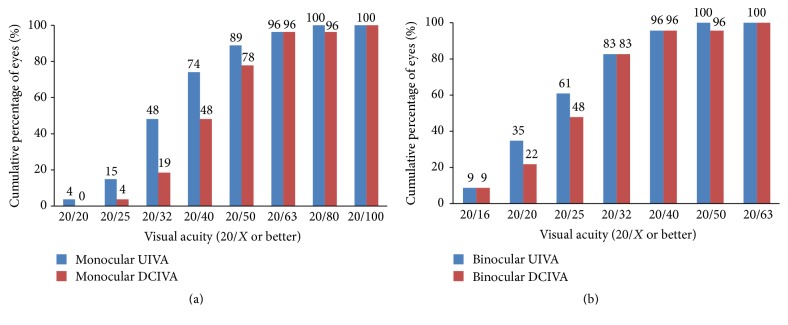
Monocular (operated eyes) and binocular uncorrected intermediate visual acuity (UIVA) and distance-corrected intermediate visual acuity (DCIVA) at 67 cm at the last visit.

**Figure 4:**
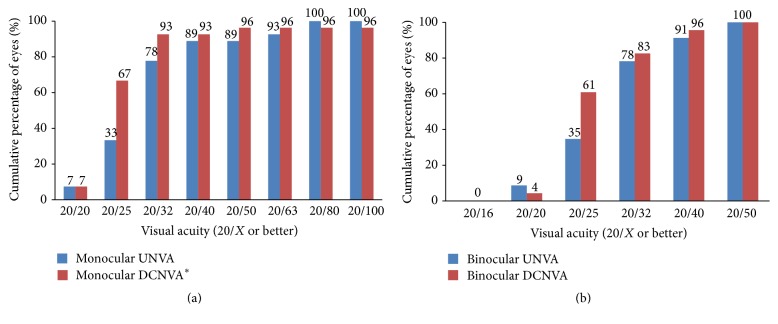
Monocular (operated eyes) and binocular uncorrected near visual acuity (UNVA) and distance-corrected near visual acuity (DCNVA) at 30 cm at the last visit. ^*∗*^One eye (4%) had DCNVA of 20/200.

Figure 1 shows the efficacy of the surgery.  Table 3 shows the mean monocular VA at different distances. In patients who underwent unilateral surgery, the UDVA, CDVA, mesopic CDVA, and UIVA of the operated eyes did not differ from the unoperated eyes (*P* > 0.05 for all comparisons); the DCIVA of the operated eyes was significantly worse than that of the unoperated eyes (*P* < 0.001); the UNVA and DCNVA of the operated eyes were significantly better than those of the unoperated eyes (*P* < 0.001 for both comparisons). Three eyes (11%) lost one line of CDVA from 20/15; no eye (0%) had more than one line of CDVA loss (Figure 1).  Table 4 shows the mean binocular VA at different distances. The DCIVA was significantly worse in the bilateral than unilateral implantation group (*P* = 0.018). Figures 2–4 show the cumulative percentages of monocular and binocular VA at different distances.

### 3.3. Contrast Sensitivity

**Figure 5:**
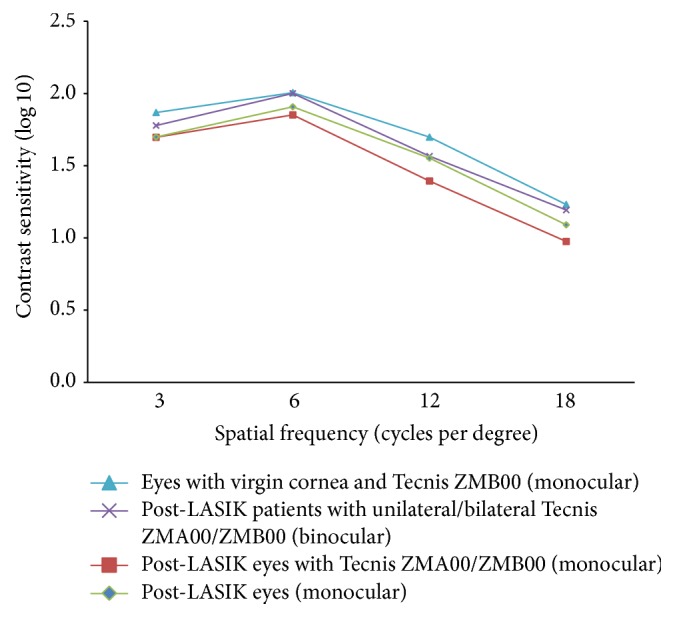
Contrast sensitivity at different spatial frequencies for postlaser in situ keratomileusis (LASIK) eyes with Tecnis ZMA00/ZMB00 implantation at the last visit (monocular) (*squares*), post-LASIK eyes at the last visit (monocular) (*diamonds*), post-LASIK patients with unilateral/bilateral Tecnis ZMA00/ZMB00 implantation at the last visit (binocular) (*crosses*), and eyes with virgin cornea and Tecnis ZMB00 implantation (monocular) (*triangles*); data from Chang et al. [4].

Figure 5 shows the monocular and binocular log CS at different spatial frequencies. Among patients who underwent unilateral surgery, the CS of the operated eyes did not differ significantly from that of the unoperated eyes (*P* > 0.05 for all comparisons). No significant difference was found between the unilateral and bilateral implantation groups (*P* > 0.05 for all comparisons).

The CS of the operated eyes at all spatial frequencies were not significantly correlated with age (*P* > 0.05 for all pairs), pre-LASIK MRSE (*P* > 0.05 for all pairs), and photopic pupillary size (*P* > 0.05 for all pairs). Linear regression showed that the mean CS at spatial frequency of 18 cpd decreased by 0.03 for every additional year of age, after adjusting for pre-LASIK MRSE and photopic pupillary size (*P* = 0.003).

### 3.4. Questionnaires

Table 5 shows the results of the VFQ-25 and supplementary questionnaire. No significant differences were found between unilateral and bilateral implantation groups in the scores in any VFQ-25 subscale items (*P* > 0.05 for all comparisons).

Thirteen (57%), 16 (70%), and nine patients (39%) reported halos, night glare, and starbursts, respectively, among whom, six (46%), 10 (63%), and three patients (33%) reported a severity score of 3 or more, respectively. The differences in the scores for all visual symptoms among the groups were not significant (*P* > 0.05 for all comparisons). Nineteen patients (83%) reported a satisfaction score of ≥3. The satisfaction score was significantly higher in bilateral implantation group (*P* = 0.021). Eighteen patients (78%) did not need spectacles at all distances; all five patients (22%) who used spectacles postoperatively underwent a unilateral implantation.

## 4. Discussion

This is the first study to report the visual outcomes, quality of vision, and quality of life after implantation of the Tecnis MIOL in patients who underwent a previous myopic LASIK procedure. A few studies have reported the distance and near VA after implantation of the ReSTOR SN60D3 and SN6AD3 (Alcon Laboratories Inc.) and AT LISA 809M MIOL (Carl Zeiss Meditec AG) in patients who underwent previous myopic [14, 16, 17] and hyperopic LASIK procedures [15, 18]. In those studies, the intermediate VA was not measured [15–17] or was presented in the form of a defocus curve without an exact value [14, 18]. The CS, binocular vision, visual symptoms, and satisfaction data were also unavailable. Since the optical design of the Tecnis MIOL differs from the ReSTOR and AT LISA MIOLs, it is also valuable to study the visual performance of the Tecnis MIOL in eyes that underwent a previous myopic LASIK procedure.

In the current study, the mean CDVA of the operated eyes of 20/19 agrees with the previous findings after LASIK (range, 20/22 to 20/20) [14–16, 18]. Under mesopic condition, the CDVA did not worsen compared with previous studies [14, 16, 18], which reported that one to two lines decrease after implantation of the spherical SN60D3 and aspheric 809M in post-LASIK eyes. MIOLs with enhanced asphericity provide better mesopic vision than those that partially correct the corneal spherical aberration (SA) [19]. With increased SA after myopic LASIK [14, 16, 27], we expected that an aspheric IOL with a more negative SA value can provide better mesopic vision. The Tecnis MIOLs correct for +0.27 *μ*m of SA [19, 28, 29] while the 809M corrects only for +0.18 *μ*m (Carl Zeiss Meditec AG, email communication, 2015) and the SN60D3 does not correct for any. This might explain the better mesopic CDVA in the current study. To retain good mesopic distance vision, eyes that underwent myopic LASIK should be implanted with an aspheric IOL and a spherical IOL for eyes treated with hyperopic LASIK [14, 16, 18].

Compared to our previous study [4] of the ZMB00 in eyes with virgin cornea, the operated eyes in the current study had significantly worse CS at spatial frequency of 3 cpd (*P* = 0.009; Mann–Whitney *U* test). This suggested that visual quality with the ZMB00 could be affected by LASIK and may be explained by the increased SA after LASIK that was not fully compensated. We found an expected negative correlation between the CS at high spatial frequency and age. However, the CS was not significantly correlated with pre-LASIK MRSE. This could be attributed to the complex interaction between partially corrected corneal SA by the MIOL, the reduction in sensitivity of the postreceptoral processes [30, 31], and morphological changes in retina [30–32] in highly myopic eyes.

For the near vision, the current mean DCNVA of 20/27 in the operated eyes was similar to the previously reported values of other bifocal MIOLs with +3- to +4-D near addition in post-LASIK eyes (range, 20/26 to 20/20) [14–16, 18] and eyes with virgin cornea (range, 20/25 to 20/20) at 30 to 40 cm [3, 4, 33]. The intermediate vision of bifocal MIOLs is weak [3–8]. The current mean DCIVA was 20/42 and significantly worse than that of the unoperated eyes. The DCIVA in eyes with virgin cornea [3–5] and defocus curves in post-LASIK eyes reported previously [14, 18] also had similar values.

The binocular DCIVA in the bilateral implantation group was significantly worse than that of the unilateral group because of the bifocal essence of the implanted MIOLs, even with binocular summation. Implanting a bifocal MIOL unilaterally in the current group of young patients preserved the intermediate vision because the unoperated eye could still accommodate [4]. Future studies with a larger sample size and comparison between age groups may be conducted to confirm this.

The postoperative refraction after MIOL implantation in post-LASIK eyes has been more unpredictable than in eyes with virgin cornea, and much effort has been made to minimize refractive surprises by obtaining corneal powers based on topography and different IOL formulas [12, 13]. Wang et al. [13] evaluated various IOL formulas from the American Society of Cataract and Refractive Society post-LASIK IOL calculator [24] and showed that formulas using no prior data and refractive change after LASIK were more accurate than the clinical history method. Abulafia et al. [34] found that the Barrett True K No History formula had significantly smaller variance in prediction error than the Shammas formula and performed similar to the Haigis-L and Modified-Masket formulas. A meta-analysis by Chen et al. [35] concluded that the clinical history method was significantly inaccurate in predicting postoperative refraction than the Haigis-L formula. The current study also had similar findings that formulas using no prior data (i.e., Barrett True K No History and Haigis-L) and refractive change after LASIK (i.e., Barrett True K) are accurate.

The binocular uncorrected VA reflects the real-life MIOL performance. The current binocular UDVA, UIVA, and UNVA were 20/20, 20/24, and 20/27, respectively. There were no significant differences at all distances between the unilateral and bilateral implantation groups but a trend of better binocular UIVA in the former group. Nevertheless, five patients (22%) still required spectacles postoperatively. They were all unilaterally operated. In one 58-year-old patient who used spectacles for all intermediate and near tasks, his binocular UIVA and UNVA were 20/38 and 20/44, respectively. The remaining four patients had good binocular UDVA (20/20), UIVA (20/24 to 20/20), and UNVA (20/34 to 20/24). They used spectacles occasionally for tasks at different distances. Since the binocular uncorrected VA at different distances were acceptable (20/34 or better) in four of the five patients who reported spectacle dependence, we believe other factors such as quality of vision and individual visual demand contributed to the spectacle dependence postoperatively.

The quality of life in patients implanted with MIOL after LASIK has not been studied previously. Overall, the VFQ-25 showed good quality of life in the current study. In the driving subscale item, the lower score (mean, 78.52) was associated with difficulty driving at night and in difficult conditions, which was consistent with a previous comparison between the Tecnis MIOLs and monofocal IOLs [26]. Nevertheless, no current patients stopped driving postoperatively.

Halos, night glare, and starbursts were common in the current study and about 50% of patients perceived them as moderate to very severe. Comparing this to our previous studies of the ZMA00 [3] and ZMB00 [4], no significant differences were found among these studies (*P* > 0.05 for all comparisons; Chi-squared test). However, more patients were dissatisfied in the current study (17%) than in our previous studies of the ZMA00 (7%) [3] and ZMB00 (4%) [4], although the difference was insignificant (*P* = 0.228; Chi-squared test). In the current study, all four dissatisfied patients had unilateral implantation and their binocular uncorrected VA at different distances were acceptable (20/30 or better). They had one or more moderate to very severe visual symptoms. On the contrary, all satisfied patients had no severe or very severe visual symptoms. For the patient who underwent LASIK enhancement, the postenhancement binocular UDVA, UIVA, and UNVA were 20/15, 20/17, and 20/19, respectively. He did not require spectacles for daily living. The patient had mild halos (score of 2.0) but was satisfied (score of 3.5).

The current study has limitations. First, we did not record the visual symptoms before MIOL implantation, which may be persistent after previous LASIK and may have contributed to the postoperative visual symptoms. Second, the sample size in the bilateral implantation group was small, so the statistical tests were underpowered for the comparisons between patients operated unilaterally and bilaterally. Third, it would be ideal to measure the HOAs to explain for the better mesopic vision in the current study than the previous one.

In conclusion, the distance and near performance after implantation of the Tecnis MIOLs after myopic LASIK were good and the intermediate vision was acceptable. Comparing to our previous studies of the same MIOLs in eyes with virgin cornea, the current CS at low spatial frequency was significantly worse but the visual symptoms were comparable.

## Figures and Tables

**Table 1:** Baseline characteristics before laser in situ keratomileusis (LASIK) and before intraocular lens implantation.

Parameters	27 operated eyes		19 unoperated eyes		*P* value^*∗*^
	Mean ± SD	Range	Mean ± SD	Range	
*Before LASIK* ^†^					
Sphere (D)	−6.03 ± 2.68	−15.00, −2.75	−6.04 ± 2.83	−13.50, −1.50	0.663
Cylinder (D)	0.90 ± 0.74	0.00, 2.25	0.66 ± 0.55	0.00, 1.75	0.484
MRSE (D)	−5.58 ± 2.67	−14.63, −2.75	−5.71 ± 2.78	−13.25, −1.50	0.740
LogMAR CDVA	−0.01 ± 0.06	−0.12, 0.10	−0.02 ± 0.07	−0.12, 0.10	1.000
*Before intraocular lens implantation*					
Sphere (D)	−0.47 ± 1.38	−5.25, 1.75	−0.26 ± 0.65	−1.75, 1.00	0.645
Cylinder (D)	0.43 ± 0.37	0.00, 1.50	0.39 ± 0.33	0.00, 1.25	0.607
MRSE (D)	−0.26 ± 1.31	−4.50, 2.13	−0.01 ± 0.65	−1.38, 1.38	0.577
LogMAR CDVA	0.01 ± 0.09	−0.12, 0.30	−0.04 ± 0.06	−0.12, 0.00	0.157
Axial length (mm)	25.59 ± 1.24	23.57, 29.77	25.50 ± 1.12	24.00, 28.69	0.498
Anterior chamber depth (mm)	3.25 ± 0.23	2.74, 3.70	3.29 ± 0.22	2.85, 3.64	0.719

^*∗*^Comparison between operated and unoperated eye in patients who underwent unilateral implantation.

^†^Three patients had LASIK in private centers elsewhere with two and three of them having no available data on pre-LASIK refraction and CDVA, respectively.

CDVA = corrected distance visual acuity; D = dioptres; LogMAR = logarithm of minimum angle of resolution; MRSE = manifest refraction spherical equivalent.

**Table 2:** Intraocular lens formula accuracy (25 eyes with pre-laser in situ keratomileusis data available).

Formula	Predicted error in refraction (dioptre)				
	Numerical		Absolute		
	Mean ± SD	Range	Mean ± SD	Median^*∗*^	Range
*Clinical history method*					
With SRK/T	0.09 ± 0.80	−2.72, 1.25	0.52 ± 0.61	0.45	0.02, 2.72
With Haigis	−1.16 ± 1.03	−5.17, 0.26	1.19 ± 0.99	1.08	0.02, 5.17
*Using Refractive Change*					
Modified-Masket	−0.55 ± 0.87	−2.55, 0.45	0.67 ± 0.77	0.41	0.03, 2.55
Barrett True K	−0.23 ± 0.85	−2.63, 0.61	0.56 ± 0.67	0.34	0.02, 2.63
*Using no prior data*					
Shammas	−0.56 ± 0.86	−3.78, 0.63	0.67 ± 0.78	0.44	0.05, 3.78
Haigis-L	−0.10 ± 0.81	−3.16, 0.09	0.49 ± 0.65	0.32	0.01, 3.16
Barrett True K No History	−0.15 ± 0.82	−3.45, 0.75	0.48 ± 0.68	0.26	0.01, 3.45

^*∗*^Comparison between clinical history method with Haigis formula and the remaining formulas (clinical history method with SRK/T, *P* = 0.001; Modified-Masket formula, *P* = 0.002; Barrett True K, *P* = 0.001; Haigis-L, *P* = 0.001; Shammas, *P* = 0.011; and Barrett True K, *P* = 0.001), where *P* < 0.002 was considered statistically significant for multiple comparisons. Comparison between the remaining formulas (*P* ≥ 0.002 for all comparisons).

SD = standard deviation.

**Table 3:** Monocular visual acuity at the last visit.

Parameter	27 operated eyes			19 unoperated eyes			*P* Value^*∗*^
	Mean Snellen equivalent	Mean ± SD (LogMAR)	Range (LogMAR)	Mean Snellen equivalent	Mean ± SD (LogMAR)	Range (LogMAR)	
UDVA	20/27	0.13 ± 0.15	0.00, 0.54	20/23	0.07 ± 0.17	−0.12, 0.40	0.197
CDVA	20/19	−0.03 ± 0.07	−0.12, 0.10	20/18	−0.05 ± 0.07	−0.12, 0.10	0.739
Mesopic CDVA	20/19	−0.02 ± 0.07	−0.12, 0.10	20/18	−0.04 ± 0.07	−0.12, 0.10	1.000
UIVA	20/32	0.22 ± 0.15	−0.03, 0.57	20/29	0.17 ± 0.22	−0.13, 0.67	0.559
DCIVA	20/42	0.32 ± 0.13	0.07, 0.47	20/26	0.11 ± 0.12	−0.07, 0.29	<0.001
UNVA	20/28	0.16 ± 0.15	−0.08, 0.58	20/64	0.51 ± 0.22	0.16, 0.82	<0.001
DCNVA	20/27	0.13 ± 0.19	−0.02, 1.02	20/70	0.55 ± 0.16	0.22, 0.74	<0.001

^*∗*^Comparison between operated and unoperated eyes in patients who underwent unilateral implantation.

CDVA = corrected distance visual acuity; DCIVA = distance-corrected intermediate visual acuity; DCNVA = distance corrected near visual acuity; LogMAR = logarithm of minimum angle of resolution; UDVA = uncorrected distance visual acuity; UIVA = uncorrected intermediate visual acuity; UNVA = uncorrected near visual acuity.

**Table 4:** Binocular visual acuity at the last visit.

Parameter	All (23 patients)			Unilateral implantation (19 patients)			Bilateral implantation (4 patients)			*P* value^*∗*^
	Mean Snellen equivalent	Mean ± SD (LogMAR)	Range (LogMAR)	Mean Snellen Equivalent	Mean ± SD (LogMAR)	Range (LogMAR)	Mean Snellen Equivalent	Mean ± SD (LogMAR)	Range (LogMAR)	
UDVA	20/20	0.00 ± 0.10	−0.12, 0.18	20/20	−0.01 ± 0.10	−0.12, 0.18	20/21	0.02 ± 0.05	0.00, 0.10	0.425
CDVA	20/17	−0.08 ± 0.07	−0.12, 0.00	20/16	−0.09 ± 0.06	−0.12, 0.00	20/19	−0.03 ± 0.10	−0.12, 0.10	0.122
Mesopic CDVA	20/17	−0.07 ± 0.07	−0.12, 0.00	20/17	−0.08 ± 0.06	−0.12, 0.00	20/20	−0.01 ± 0.09	−0.12, 0.10	0.094
UIVA	20/24	0.08 ± 0.13	−0.13, 0.33	20/23	0.07 ± 0.11	−0.13, 0.27	20/30	0.17 ± 0.16	−0.03, 0.33	0.165
DCIVA	20/25	0.10 ± 0.13	−0.13, 0.43	20/24	0.07 ± 0.11	−0.13, 0.29	20/36	0.25 ± 0.13	0.13, 0.43	0.018
UNVA	20/27	0.13 ± 0.10	−0.08, 0.34	20/26	0.12 ± 0.10	−0.08, 0.34	20/29	0.16 ± 0.11	0.08, 0.32	0.684
DCNVA	20/26	0.11 ± 0.08	−0.02, 0.32	20/26	0.11 ± 0.08	−0.02, 0.32	20/26	0.11 ± 0.12	0.02, 0.28	0.775

^*∗*^Comparison between unilateral and bilateral implantation.

CDVA = corrected distance visual acuity; DCIVA = distance-corrected intermediate visual acuity; DCNVA = distance corrected near visual acuity; LogMAR = logarithm of minimum angle of resolution; UDVA = uncorrected distance visual acuity; UIVA = uncorrected intermediate visual acuity; UNVA = uncorrected near visual acuity.

**Table 5:** Results for the National Eye Institute Visual Functioning Questionnaire-25 and supplementary questionnaire at the last visit (23 patients).

Parameter	Mean equivalent description	Mean ± SD	Range
*National Eye Institute Visual Functioning Questionnaire-25* ^*∗*^			
General health	Good/very good	63.04 ± 16.63	25.00, 100.00
General vision	Fair/good	68.70 ± 10.14	60.00, 80.00
Ocular pain	None/a little of the time; none/mild	79.89 ± 20.09	25.00, 100.00
Near activities	No difficulty at all/a little difficulty	92.25 ± 10.21	70.00, 100.00
Distance activities	No difficulty at all/a little difficulty	92.32 ± 14.53	33.33, 100.00
Vision specific:			
Social functioning	No difficulty at all/a little difficulty	98.37 ± 4.30	87.50, 100.00
Mental health symptoms	None/a little of the time; mostly/definitely false	90.49 ± 14.70	43.75, 100.00
Role difficulties	Mostly/definitely false	90.76 ± 16.95	50.00, 100.00
Dependency on others	Mostly/definitely false	94.20 ± 17.12	33.33, 100.00
Driving^†^	No difficulty at all/a little difficulty	78.52 ± 19.28	33.33, 100.00
Color vision	No difficulty at all	100.00 ± 0.00	100.00, 100.00
Peripheral vision	No difficulty at all	100.00 ± 0.00	100.00, 100.00
Composite	—	90.31 ± 8.50	68.29, 98.00
*Visual symptoms* ^‡^			
Halos	Very mild/mild	1.46 ± 1.62	0.0, 5.0
Night glare	Very mild/mild	1.85 ± 1.69	0.0, 5.0
Starbursts	None/very mild	0.78 ± 1.31	0.0, 5.0
*Satisfaction* ^§^	Neutral/satisfied	3.50 ± 1.02	1.0, 5.0
*Number of patients (%) who would undergo the surgery again*	17 (74)		
*Number of patients (%) who would recommend the surgery to their friends or relatives*	17 (74)		
*Number of patients (%) who did not use spectacles for*			
Distance tasks	22 (96)		
Intermediate tasks	20 (87)		
Near tasks	20 (87)		
Any distances	18 (78)		

^*∗*^Score on a 0 to 100 scale.

^†^Nine patients were current or ever driver.

^‡^Level of visual symptoms (0, none; 1, very mild; 2, mild; 3, moderate; 4, severe; 5, very severe).

^§^Level of satisfaction (1, very dissatisfied; 2, dissatisfied; 3, neutral; 4, satisfied; 5, very satisfied).
